# Autoantibodies against Modified Histone Peptides in SLE Patients Are Associated with Disease Activity and Lupus Nephritis

**DOI:** 10.1371/journal.pone.0165373

**Published:** 2016-10-25

**Authors:** Jürgen Dieker, Jo H. Berden, Marinka Bakker, Jean-Paul Briand, Sylviane Muller, Reinhard Voll, Christopher Sjöwall, Martin Herrmann, Luuk B. Hilbrands, Johan van der Vlag

**Affiliations:** 1 Department of Nephrology, Radboud Institute for Molecular Life Sciences, Radboud University Medical Center, Nijmegen, The Netherlands; 2 CNRS, Immunopathologie et chimie thérapeutique/Laboratory of excellence MEDALIS, Institut de Biologie Moléculaire et Cellulaire, Strasbourg, France; 3 Department of Rheumatology and Clinical Immunology, and the Center for Chronic Immunodeficiency, University Medical Center Freiburg, Freiburg/Breisgau, Germany; 4 Rheumatology/AIR, Department of Clinical and Experimental Medicine, Linköping University, Linköping, Sweden; 5 Department for Internal Medicine 3, University Hospital Erlangen, Friedrich-Alexander University of Erlangen-Nuremberg, Erlangen, Germany; Universite Paris-Sud, FRANCE

## Abstract

Persistent exposure of the immune system to death cell debris leads to autoantibodies against chromatin in patients with systemic lupus erythematosus (SLE). Deposition of anti-chromatin/chromatin complexes can instigate inflammation in multiple organs including the kidney. Previously we identified specific cell death-associated histone modifications as targets of autoantibodies in SLE. In this study we addressed, in a large cohort of SLE patients and controls, the question whether plasma reactivities with specific histone peptides associated with serology and clinical features. Plasma from SLE patients with and without lupus nephritis, disease controls, and healthy controls, were tested in ELISA with histone H4 peptide acetylated at lysines 8, 12 and 16 (H4p^ac^), H2B peptide acetylated at lysine 12 (H2Bp^ac^), H3 peptide trimethylated at lysine 27 (H3p^me^), and their unmodified equivalents. SLE patients displayed a higher reactivity with the modified equivalent of each peptide. Reactivity with H4p^ac^ showed both a high sensitivity (89%) and specificity (91%) for SLE, while H2Bp^ac^ exhibited a high specificity (96%) but lower sensitivity (69%). Reactivity with H3p^me^ appeared not specific for SLE. Anti-H4p^ac^ and anti-H2Bp^ac^ reactivity demonstrated a high correlation with disease activity. Moreover, patients reacting with multiple modified histone peptides exhibited higher SLEDAI and lower C3 levels. SLE patients with renal involvement showed higher reactivity with H2B/H2Bp^ac^ and a more pronounced reactivity with the modified equivalent of H3p^me^ and H2Bp^ac^. In conclusion, reactivity with H4p^ac^ and H2Bp^ac^ is specific for SLE patients and correlates with disease activity, whereas reactivity with H2Bp^ac^ is in particular associated with lupus nephritis.

## Introduction

Autoantibodies in patients with SLE are directed against numerous nuclear constituents including chromatin [1–2], for which the basic structure consists of DNA wrapped around a core of histone proteins H2A, H2B, H3 and H4. Approximately 40% of the SLE patients develop lupus nephritis, where inflammation occurs in the filtration units of the kidney, i.e. the glomeruli, due to the deposition of chromatin/anti-chromatin complexes [2,3]. Instigation of the anti-chromatin response in SLE is explained by an insufficient removal of cells undergoing programmed cell death (apoptosis) or neutrophil extracellular trap formation (NETosis) [4,5]. This leads to the persistent presence of chromatin-containing material derived from death cells as we have recently shown in SLE patients [6]. The immunostimulatory properties of death cell-derived chromatin are enhanced by apoptosis-related post-translational modifications (PTMs) of the N-terminal tails of histones [7]. Characterization of the epitopes of monoclonal autoantibodies derived from lupus mice previously led to the identification of several apoptosis-associated histone modifications, including histone H4 acetylation at lysines 8, 12 and 16 [8], H2B acetylation at lysine 12 [9], and H3 trimethylation at lysine 27 [10]. The identified histone modifications are also present in non-apoptotic cells, where these have been linked to processes such as the regulation of gene expression. However, we have previously shown that the amount of these modifications hugely increases when cells go into apoptosis or NETosis [8–11]. Importantly, autoantibodies in plasma samples of murine lupus models and SLE patients recognized histone peptides with these identified PTMs more avidly compared to the corresponding unmodified peptides [8–10]. In addition, circulating apoptotic particles in SLE patients contain chromatin with these apoptosis-associated histone modifications [6]. Recently, we have shown that these histone modifications are also increased in neutrophil extracellular traps (NETs) [11]. SLE plasma also contain autoantibodies that recognize several additional types of chromatin PTMs, like peroxynitrite-treated H2A [12], isomerized H2B [13], and conformational acetylated epitopes [14].

Peptides displaying PTMs are powerful tools for the diagnosis of autoimmune diseases in which post-translationally modified autoantigens play a pathogenic role [15]. Assays using peptides that contain citrulline (deiminated arginine residues) are highly predictive and specific for the diagnosis of rheumatoid arthritis [16]. Recently, two distinct subfamilies of anti-citrulline antibodies against citrullinated fibrin-derived peptides were identified [17]. In addition, two citrullinated histone H4 peptides were shown to be preferentially recognized by autoantibodies present in the majority of patients with rheumatoid arthritis, but not in SLE [18]. Another example is the use of N-glycosylated peptides as an antigenic biomarker for patients with multiple sclerosis [19]. The aforementioned studies also suggest that reactivity to specific modified epitopes can be related to different autoimmune disease manifestations. With regard to SLE, the diagnostic potential of peptides that contain PTMs remains rather elusive at present. Total chromatin and/or dsDNA are the primary antigens employed in diagnostic assays, including ELISA, and Farr and Crithidia assays, while histones are used to a lesser extent. In the past, several histone peptides comprised of different, mostly N-terminal, regions of histones have been identified as targets for autoantibodies in SLE patients, but these studies did not include modified residues [20–21]. Although, assays to detect antibodies against chromatin or dsDNA in SLE display a relatively high specificity and sensitivity, in particular in patients with lupus nephritis [2,22–23], they also have some drawbacks. The exact composition of the chromatin in the assay largely depends on the source used for isolation, and chromatin modifications obviously vary extensively between different cells or tissues. This might explain why different studies reported a high variability in specificity and sensitivity when using different anti-dsDNA and anti-chromatin assays [2, 23]. More importantly, in these cases chromatin/dsDNA was isolated from sources that mostly consist of healthy, non-apoptotic cells. Therefore, the isolated chromatin will not possess the same type and amount of PTMs compared to chromatin derived from apoptotic cells. Therefore, the antigens normally used in the assay do not represent the actualautoantigens responsible for the induction of the autoimmune response in SLE patients. Two recent studies, that used advanced microarray-based assays, have underlined the potential impact of modified histone peptides for the diagnosis of SLE [24–25]. These studies compared the reactivity of sera from a selected group of SLE patients with a range of different modified and unmodified histone peptides. An enhanced reactivity was observed with several modified histone peptides compared to the corresponding peptides without the modifications. Importantly, the modifications we previously linked to SLE, i.e. histone H4 peptides acetylated at lysines 8, 12 and 16, H3 peptides trimethylated at lysine 27 and H2B acetylated at lysine 12 were among the prime targets for SLE autoantibodies as reported by aforementioned studies. The highest reactivity was found in SLE patients with a prominent IFN-signature, which is associated with higher disease activity [24].

In this study we measured the reactivity with our previously identified apoptosis modified histone peptides in a large cohort of SLE patients with and without nephritis, disease controls and healthy controls, and evaluated the correlation with serology and clinical parameters.

## Methods

### Patients

Cross-sectional plasma from 102 SLE patients with active, biopsy-proven proliferative lupus nephritis (formerly known as WHO class III, IV, Vc or Vd), 76 SLE patients without nephritis, consecutive sera from 15 SLE patients experiencing disease flares, as well as plasmas from 12 patients with rheumatoid arthritis (RA) and 12 patients with systemic sclerosis (SSc) were collected at the Radboud University Medical Center, the Rheumatology Clinic at Linköping University Hospital, and the University Hospital Erlangen. Plasma samples were taken at the time of diagnosis. The majority of patients were Caucasian and female, and all patients with SLE fulfilled ≥4 American College of Rheumatology (ACR) criteria [26]. Disease activity was measured with total SLE disease activity index (SLEDAI) [27], or SLEDAI-2K [28]. Healthy controls used in this study were age- and gender matched. Oral and written informed consent was obtained from all subjects. The study protocol was approved by the Linköping University Ethical Review Board, Sweden; by the Radboud University Medical Centre Ethical Review Board, the Netherlands; and by the University Hospital Erlangen Ethical Review Board, Germany.

### ELISA and Other Immunoassays

Peptides used for coating were either synthesized using in-house facilities in Strasbourg or obtained commercially (Abcam, Cambridge, U.K.) and are listed in Table 1. Two μM of peptide was coated overnight, blocked with 5% fetal calf serum (FCS) in PBS, incubated for 2 hours with plasma diluted 1:50 and 1:100, and subsequently incubated for 1 hour with horseradish peroxidase (HRP)–conjugated goat anti-human IgG(H+L) diluted 1:10,000 (Southern Biotechnology, Birmingham, U.S.A.), as previously described.[5] Each patients’ plasma was tested with all peptides in the same experiment. Reactivity with each peptide was calculated as OD (450nm) x dilution. Cut-off values were determined as 3x background signal (without plasma) and were set at 12.6 (H4p), 12.7 (H4p^ac^), 11.7 (H2Bp), 14.7 (H2Bp^ac^), 14.9 (H3p) and 16.9 (H3p^me^). Peptide coating was checked by incubation with the appropriate lupus mouse-derived monoclonal antibodies (KM-2, LG11-2, or BT164) [8–10]. Anti-histone, -dsDNA,–chromatin, and –C1q ELISAs were performed as previously described [29]. Briefly, for the anti-dsDNA ELISA, calf thymus dsDNA (Roche, Almere, The Netherlands) was coated overnight in phosphate-buffered saline (20 mg/ml). The anti-nucleosome ELISA, was performed using calf thymus H1-stripped chromatin (kindly provided by Dr R Burlingame, INOVA Diagnostics Inc, San Diego, California, USA), diluted in phosphate-buffered saline (5 mg/ml). In the anti-histone ELISA, calf thymus total histones (containing histones H1, 2A, 2B, 3 and 4; Roche) were coated overnight (2.5 mg/ml) in 0.1 M glycine buffer at pH 9. Rest of the assay was performed as described above for peptides. For the anti-C1q ELISA, plates were coated overnight with 3 g/well plasma-derived C1q in bicarbonate buffer pH 9.6. Calf thymus-derived chromatin and total histones tested positive for monoclonal antibodies against non-modified and modified histones [8–10]. Measurements of anti-dsDNA antibodies in Crithidia and Farr assays were performed according to local standards, as previously described [30,31].

**Table 1:** Histone peptides used in this study.

Abbreviation^1^	Peptide	Sequence
H4p	H4^1-22^	SGRGKGGKGLGKGGAKRHRKVL
H4p^ac^	H4^1-22^-K8,12,16ac	SGRGKGGK(Ac)GLGK(Ac)GGAK(Ac)RHRKVL
H2Bp	H2B^1-18^	PDPAKSAPAPKKGSKKAV
H2Bp^ac^	H2B^1-18^-K12ac	PDPAKSAPAPKK(Ac)GSKKAV
H3p	H3^18-37^	KQLATKVARKSAPATGGVKK
H3p^me^	H3^21-34^-K27me3	ATKVARK(me3)SAPATGG

^1^H4p, histone H4 peptide; H4p^ac^, histone H4 peptide acetylated at lysine 8, 12 and 16; H2Bp, histone H2B peptide; H2Bp^ac^, histone H2B peptide acetylated at lysine 12; H3p, histone H3 peptide; H3p^me^, histone H3 peptide trimethylated at lysine 27

### Statistics

**Fig 1:**
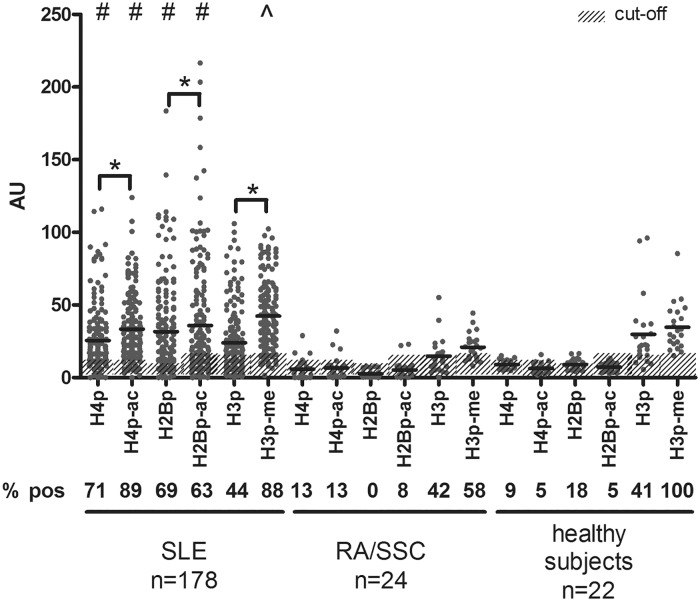
Reactivity with histone peptides containing apoptosis-associated modifications and their unmodified counterparts. Plasma samples from SLE patients were tested in ELISA with the indicated modified histone peptides and their unmodified counterparts (i.e. H4p, H2Bp, and H3p). Patients with rheumatoid arthritis (RA) or systemic sclerosis (SSc), and healthy individuals were used as controls. * p<0.01 compared to the unmodified equivalent as determined using the Mann-Whitney U test; # p<0.01 compared to healthy subjects and patients with other autoimmune diseases and ^ p<0.01 compared to patients with other autoimmune diseases using the one-way ANOVA test. H4p, histone H4 peptide; H4p^ac^, histone H4 peptide acetylated at lysine 8, 12 and 16; H2Bp, histone H2B peptide; H2Bp^ac^, histone H2B peptide acetylated at lysine 12; H3p, histone H3 peptide; H3p^me^, histone H3 peptide trimethylated at lysine 27.

**Fig 2:**
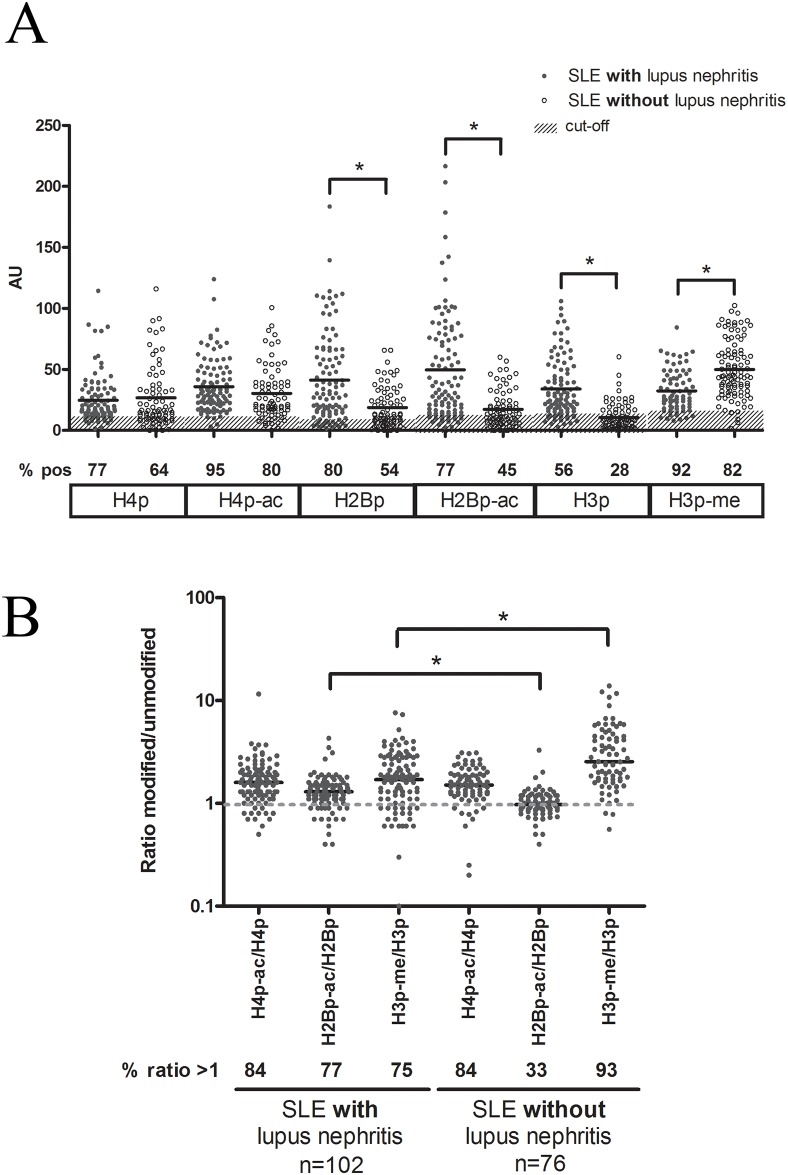
Reactivity with histone peptides containing apoptosis-associated modifications and with their unmodified counterparts in SLE patients with and without lupus nephritis. (**A**) Plasma samples from SLE patients with or without nephritis were tested in ELISA with the indicated modified histone peptides and their unmodified counterparts (i.e. H4p, H2Bp, and H3p). (**B**) Ratio of the reactivity with the respective modified histone peptide divided by the reactivity with the unmodified counterpart, for SLE patients with and without nephritis. In all cases the one-way ANOVA test was used for statistical comparison. * p<0.001. H4p, histone H4 peptide; H4p^ac^, histone H4 peptide acetylated at lysine 8, 12 and 16; H2Bp, histone H2B peptide; H2Bp^ac^, histone H2B peptide acetylated at lysine 12; H3p, histone H3 peptide; H3p^me^, histone H3 peptide trimethylated at lysine 27.

Statistical analysis was performed using SPSS (Chicago, Illinois, U.S.A.) and Graphpad Prism (San Diego, California, U.S.A.). The one-way ANOVA test was used for comparison of the reactivity between multiple groups (Figs 1 and 2B, and S2 Table), and p values were corrected using the Bonferroni’s Multiple Comparison Test. ROC curves were compared using the Delong method. In addition, the Mann-Whitney U test was used for comparison of two individual groups (Fig 2A), and the Wilcoxon signed rank test was used to compare sets of ordinal data (S2 Table). Spearman’s rank correlation coefficient was used to compare correlations. P values <0.05 were regarded as significant, unless stated otherwise.

## Results

### Reactivity with H4p^ac^ and H2Bp^ac^ Is Specific for SLE patients, whereas H2Bp^ac^ Reactivity Is Particularly Associated with Lupus Nephritis

A cohort of 178 patients with SLE was tested in ELISA for reactivity with H4p^ac^, H2Bp^ac^ and H3p^me^, along with their unmodified equivalents (Table 1). The percentage of positive SLE patients was highest for H4p^ac^ and H3p^me^, followed by H4p, H2p^ac^ and H2Bp, and lowest for H3p (Fig 1). Overall, SLE patients demonstrated a significantly higher reactivity with the modified histone peptide, as compared with the unmodified equivalent. Within the cohort, enhanced reactivity with the modified equivalent was observed in 84%, 58% and 83% of the patients for H4p^ac^, H2B^ac^ and H3p^me^, respectively. SLE patients showed a significantly higher reactivity with H4p, H4p^ac^, H2Bp and H2Bp^ac^, compared to patients with other autoimmune disease (RA or SSc), and healthy subjects (Fig 1). For H3p, no significant difference was observed, while H3p^me^ reactivity was only significantly higher compared to patients with other autoimmune diseases. In fact, patients with RA or SSc tested only occasionally positive for H4P (3/24), H4p^ac^ (3/24), H2Bp (0/24), or H2Bp^ac^ (2/24), while a considerable number of these patients tested positive for H3p (11/24) and H3p^me^ (15/24). Higher reactivity of RA/SScplasmas with modified peptides was sporadically found for H4p^ac^ and H2Bp^ac^ (1/24 and 2/24 respectively), but more frequently for H3p^me^ (9/24). There was no significant difference between RA and SSc patients. Overall the reactivity with modified peptides was not significantly different from that with the unmodified equivalents for RA and SSc. Samples of healthy subjects also tested occasionally positive for H4P, H4p^ac^, H2Bp or H2Bp^ac^, but the reactivity was always rather low (<20 AU, Fig 1) and no superior reactivity with modified peptides was found. Surprisingly, we found that the majority of healthy controls tested positive for H3p and H3p^me^.

Within the cohort of SLE patients, patients with and without lupus nephritis were compared (Fig 2). For H4p and H4p^ac^, a higher percentage of positives in patients with lupus nephritis was observed, while the median reactivity (Fig 2a) and modified/unmodified ratio (Fig 2b) was similar between both groups. For H2p and H2p^ac^ SLE patients without lupus nephritis exhibited a lower percentage of positives, a lower median reactivity and a lower modified/unmodified ratio. In addition, patients without lupus nephritis showed a lower reactivity with H3p, a higher reactivity with H3p^me^, and as a consequence a higher ratio of H3p^me^/H3p. For 49% of the SLE patients with lupus nephritis, versus 28% for patients without lupus nephritis, an enhanced reactivity with the modified equivalent for all three modified histone peptides was observed. In addition, the majority of patients with lupus nephritis tested positive for three modified peptides (75%), while 20% tested positive for 2 modified peptides, 3% for 1 modified peptide, and only 2% was negative for all of the modified peptides. Patients that tested negative for H4p^ac^, also tested negative for H2Bp^ac^, while only part of those patients were negative for H3p^me^. For patients without lupus nephritis, 35%, 46% and 11% tested positive for three, two and one modified peptide, respectively.

**Fig 3:**
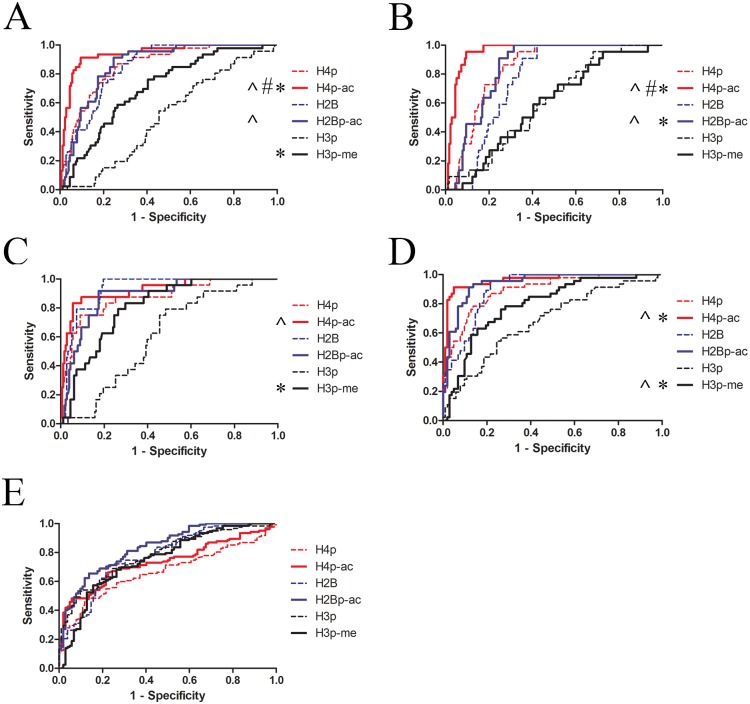
H4p^ac^ appears superior in distinguishing SLE patients from healthy and disease controls. ROC curves for (modified) histone peptide ELISAs were calculated for SLE vs. all controls (**A**), SLE vs. healthy controls (**B**), SLE vs. disease controls (**C**), lupus nephritis vs. all controls (not including SLE without nephritis) (**D**), and lupus nephritis vs. all controls including SLE without nephritis (**E**). *, p<0.05 vs. unmodified; #, p<0.01 vs. H2Bp^ac^; ^, p<0.01 vs. H3p^me^.

Sensitivity and specificity were determined for each of the tested histone peptides (Table 2), and receiver operator characteristics (ROC) curves were calculated (Fig 3). In all cases the sensitivity for the modified peptide was higher compared to the unmodified equivalent, which was also reflected in the ROC curves (Fig 3a–3c). As shown in Table 2, H4p^ac^ showed a very high sensitivity (88.8%) and specificity (91.3%, compared to all controls), while H2Bp^ac^ showed a high specificity (95.7%) but lower sensitivity (69.1%) and H3p^me^ showed a high sensitivity (87.6%) but very low specificity (20.0%). H4p^ac^ appeared to be the best performer in identifying SLE patients when compared to all controls or healthy controls (Fig 3a and 3c), followed by H2Bp^ac^, while differences were smaller when SLE patients were only compared to disease controls (Fig 3b). Specificity for H4p^ac^ and H2Bp^ac^ was similar when compared to healthy controls and disease controls (Table 2). Combining reactivity with different modified peptides did not significantly improve sensitivity and/or specificity. Sensitivity for all peptides, and in particular for H4p/H4p^ac^ and H2Bp/H2Bp^ac^ significantly increased when only SLE patients with lupus nephritis were included in the calculations. Finally, the specificity for H4p^ac^ and H2Bp^ac^ of patients with lupus nephritis versus all controls including SLE patients without lupus nephritis, was 46.7% and 67.2%, respectively. ROC curves in Fig 3d and 3e confirmed that comparing SLE patients, including patients with nephritis, with healthy controls and RA/SSc patients resulted in a high area under the curve for H4p^ac^ and H2Bp^ac^, while a lower area under the curve was observed when lupus nephritis patients were compared with all controls including SLE patients without nephritis.

**Table 2:** Specificity and sensitivity of (modified) histone peptide ELISAs in SLE patients.

	SLE	Lupus nephritis	SLE vs. all controls	SLE vs. healthy controls	SLE vs. disease controls	Lupus nephritis vs. all controls incl. SLE w/o nephritis
Peptide^1^	Sensitivity (%)	Sensitivity (%)	Specificity (%)	Specificity (%)	Specificity (%)	Specificity (%)
H4p^ac^	88.8	95.0	91.3	87.5	95.5	46.7
H4p	49.4	78.2	87.0	87.5	86.4	55.7
H2Bp^ac^	69.1	77.4	95.7	91.7	100	67.2
H2Bp	63.4	80.4	91.3	100	77.3	63.1
H3p^me^	87.6	92.2	20.0	37.5	4.6	19.7
H3p	43.8	55.9	58.7	58.3	19.1	67.2
H4p^ac^ or H2Bp^ac^	90.4	95.0	87.0	79.2	95.5	42.6
H4p^ac^ and H2Bp^ac^	66.3	85.2	100	100	100	74.6

^1^H4p, histone H4 peptide; H4p^ac^, histone H4 peptide acetylated at lysine 8, 12 and 16; H2Bp, histone H2B peptide; H2Bp^ac^, histone H2B peptide acetylated at lysine 12; H3p, histone H3 peptide; H3p^me^, histone H3 peptide trimethylated at lysine 27

### Reactivity with H4p^ac^ and H2Bp^ac^ Correlates with Disease Activity in SLE Patients

The correlation of reactivities with different peptides, other nucleosomal antigens, and several relevant clinical parameters was determined for SLE patients with lupus nephritis. The reactivity towards each modified peptide highly correlated with the reactivity against the unmodified equivalent (Table 3). For 61 patients the reactivity in ELISA with dsDNA, chromatin, histones, and C1q; and the reactivity with anti-dsDNA antibodies in routine diagnostic assays (Farr and Crithidia assay) were measured. Anti-H4p^ac^, anti-H2Bp^ac^ and anti-H2Bp reactivity was highly correlated with anti-dsDNA and anti-chromatin reactivity, while reactivity with H4p, H3p^me^ or H3p showed a low correlation with anti-dsDNA and no correlation with anti-chromatin reactivity. Only the reactivity with H2Bp, H2Bp^ac^ and H3p significantly correlated with anti-histone reactivity. Anti-H2Bp^ac^, H2Bp and H3p showed a significant correlation with the Farr assay. Weak or absent correlations were observed between peptide reactivities and C1q, and the Crithidia assay.

**Table 3:** Correlation coefficients between reactivity with modified and unmodified histone peptides, and other serological assays in patients with lupus nephritis.

Assay^1^	H4p ac		H4p		H2Bp ac		H2Bp		H3p me		H3p	
		p		p		p		p		p		p
H4p^ac^	-	-	-	-	-	-	-	-	-	-	-	-
H4p	**0.75**	<0.001	-	-	-	-	-	-	-	-	-	-
H2Bp^ac^	**0.54**	<0.001	**0.46**	0.02	-	-	-	-	-	-	-	-
H2Bp	**0.49**	<0.001	**0.38**	0.03	**0.94**	<0.001	-	-	-	-	-	-
H3p^me^	**0.58**	<0.001	**0.53**	<0.001	**0.38**	0.01	**0.35**	<0.001	-	-	-	-
H3p	**0.42**	0.001	**0.38**	0.024	**0.52**	0.01	**0.55**	<0.001	**0.48**	0.001	-	-
dsDNA	**0.50**	<0.001	**0.34**	0.007	**0.60**	<0.001	**0.59**	<0.001	**0.27**	0.036	**0.42**	0.001
Chromatin	**0.34**	0.007	0.22	0.091	**0.40**	0.001	**0.40**	0.002	0.10	0.430	0.28	0.078
Histone	0.25	0.055	0.14	0.273	**0.44**	<0.001	**0.52**	<0.001	0.18	0.076	**0.38**	0.011
C1q	0.20	0.120	0.20	0.121	0.13	0.318	0.13	0.337	0.21	0.111	0.13	0.313
Farr	0.23	0.101	0.21	0.140	**0.44**	0.001	**0.41**	0.002	0.18	0.208	**0.38**	0.006
Crithidia	0.10	0.470	0.14	0.277	**0.27**	0.290	**0.29**	0.038	0.08	0.540	0.25	0.056

^1^H4p, histone H4 peptide; H4p^ac^, histone H4 peptide acetylated at lysine 8, 12 and 16; H2Bp, histone H2B peptide; H2Bp^ac^, histone H2B peptide acetylated at lysine 12; H3p, histone H3 peptide; H3p^me^, histone H3 peptide trimethylated at lysine 27

**Fig 4:**
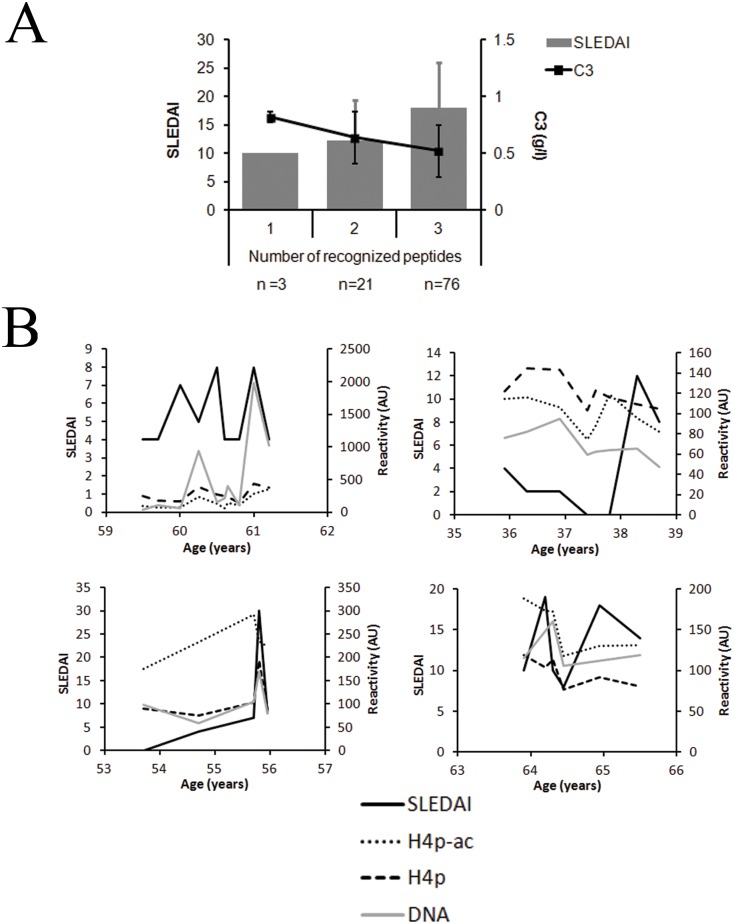
Reactivity with (multiple) modified peptides is associated with increased SLEDAI and follow disease activity during disease flares. **(A)** SLE patients with nephritis were sorted into groups depending on the number of different modified histone peptides they show reactivity with, and the mean SLEDAI and C3 levels were compared between these groups. (**B**) Examples of reactivity with H4p^ac^ and H4p along with the SLEDAI score and anti-dsDNA reactivity in 4 patients that experienced a disease flare after initial treatment. Patient characteristics are detailed in S1 Table. H4p, histone H4 peptide; H4p^ac^, histone H4 peptide acetylated at lysine 8, 12 and 16; H2Bp, histone H2B peptide; H2Bp^ac^, histone H2B peptide acetylated at lysine 12; H3p, histone H3 peptide; H3p^me^, histone H3 peptide trimethylated at lysine 27

The correlation of reactivity with modified histone peptides with markers for disease activity was also determined in SLE patients with nephritis (Table 4). Anti-H4p^ac^ reactivity significantly correlated with SLEDAI and inversely with C3 levels, while H4p did not show any significant correlation. Anti-H2Bp^ac^ and H2Bp reactivity both showed a significant correlation with SLEDAI and a significant inverse correlation with C3 levels. In contrast, anti-H3p^me^ and H3 reactivity did not correlate with SLEDAI or C3 levels. Reactivity with dsDNA, chromatin or histones tested in ELISA and anti-dsDNA reactivity quantified by the Farr assay, also correlated with the SLEDAI for these patients. Only anti-dsDNA and Farr showed an inverse correlation with C3. Interestingly, patients who demonstrated reactivity with multiple modified histone peptides displayed an increase of the SLEDAI and a decrease in C3 levels (Fig 4a). No significant correlation of anti- H2p^ac^ and anti-H4p^ac^ reactivity with standard parameters for renal function were found, although a trend towards a higher incidence of sustained doubling of creatinine, or relapse in H2Bp^ac^-high patients could be observed (S1 Table, see Additional file 1.pdf). Time courses of plasma’s from SLE patients covering pre-flare, the flare and the post-flare periods are rather unique. However, in 11 patients who experienced a disease flare after initial treatment, and of whom 4 representative patients (S2 Table, see Additional file 2.pdf) are shown in Fig 4b, a higher reactivity with H4p^ac^ could be demonstrated, followed, or sometimes even preceded, an increase in SLEDAI and/or anti-dsDNA reactivity. No correlation of reactivity with the respective modified histone peptide with a specific disease manifestation during the flare was observed.

**Table 4:** Correlation coefficients between reactivity with modified and unmodified histone peptides, dsDNA, chromatin or histones, and disease activity (SLEDAI) or complement C3 levels in SLE patients with lupus nephritis.

Assay^1^	SLEDAI		C3	
		p		p
H4p^ac^	**0.42**	0.01	**-0.29**	0.023
H4p	0.25	0.056	-0.11	0.390
H2Bp^ac^	**0.45**	<0.0001	**-0.37**	0.003
H2Bp	**0.44**	0.002	**-0.37**	0.003
H3p^me^	0.25	0.055	0.08	0.547
H3p	0.14	0.304	-0.22	0.091
dsDNA	**0.42**	<0.0001	**-0.41**	<0.0001
Chromatin	**0.29**	<0.0001	-0.23	<0.0001
Histone	**0.36**	<0.0001	-0.24	<0.0001
Farr	**0.26**	<0.0001	**-0.31**	<0.0001
Crithidia	0.01	<0.0001	-0.19	<0.0001

^1^H4p, histone H4 peptide; H4p^ac^, histone H4 peptide acetylated at lysine 8, 12 and 16; H2Bp, histone H2B peptide; H2Bp^ac^, histone H2B peptide acetylated at lysine 12; H3p, histone H3 peptide; H3p^me^, histone H3 peptide trimethylated at lysine 27

## Discussion

Our study revealed a strong reactivity with multiple modified histone peptides in the majority of SLE patients with a sensitivity of ~90% for H4p^ac^, and a specificity >90% for H4p^ac^, and H2Bp^ac^. The reactivity with H4p/H4p^ac^ and H2Bp/H2Bp^ac^ observed in this study was similar to previous results obtained in much smaller cohorts of controls and SLE patients [8–9]. In the majority of the SLE patients, the reactivity with the modified peptides was increased by 25–100% compared to the unmodified peptide. Reactivity with H3p^me^ showed a high sensitivity, but demonstrated no specificity for patients with SLE. The unexpected lack of specificity for anti-H3p/H3p^me^ reactivity in our assay might be due to a decreased accessibility within the circulating nucleosome/apoptotic microparticle, for which we found evidence in a previous study [6]. Another explanation might be the reactivity against methylated residues in plasmas from healthy individuals, which has been described before for IgM [32]. Importantly, we demonstrated that the modified peptides H4p^ac^ and H2Bp^ac^ show an increased specificity and sensitivity compared to their unmodified counterpart. In addition, a higher correlation with disease activity, before treatment and during disease flares, was observed for H4p^ac^ and H2Bp^ac^. Therefore, these modified peptides appear to have a higher diagnostic value compared to their unmodified counterparts. We have recently shown that circulating histones and apoptotic microparticles, and *in vitro* generated NETs from SLE patients contain aforementioned histone modifications, and that these modified histones, apoptotic microparticles and NETs are very potent to stimulate the immune system [6,8,11]. Aforementioned histone modifications also occur in non-apoptotic cells, but in much lower amounts [8–11]. Consequently non-apoptotic cell-derived chromatin or microparticles have a significant lower immunostimulatory capacity, as we previously have shown [6,8,11]. Moreover, others have used a large screen of different modified and unmodified histone peptides, and identified our original panel of histone modifications among the most prominent targets for autoantibodies in SLE patients [24,25]. Therefore, our results also confirm the important underlying role of cell death-modified histones in the development of SLE.

Reactivity with all (modified) histone peptides that were tested showed a significant correlation with anti-dsDNA reactivity. However, anti-H3p^me^/H3p reactivity did not correlate with anti-chromatin, while anti-H4p^ac^/H4p and anti-H3p^me^ reactivity did not correlate with overall anti-histone reactivity. Importantly, chromatin and histones used in the respective assays were isolated from calf thymus, which contains only a low amount of apoptotic cells and therefore, a relatively low amount of the (apoptosis- and NETosis-associated) histone modifications. Indeed, in our hands calf thymus-derived chromatin and histones show reactivity with monoclonal antibodies directed against modified histones, but this reactivity is much lower compared to histones/chromatin derived from apoptotic cells. In addition, non-apoptotic histone possess other modifications in the same regions, which might interfere with the binding of the autoantibodies we measure in our anti-peptide assays Therefore, the difference in the type and amount of histone modifications can explain the lack of correlation that we found between the reactivity with the panel of modified and unmodified histone peptides, and non-apoptotic chromatin and histones. We previously observed a sensitivity of 81% for anti-chromatin and 96% for anti-dsDNA in the same cohort of SLE patients with nephritis [29]. Other studies reported different sensitivities and specificities for anti-chromatin (ranging from 45–100% and 88–100%, respectively), and for anti-dsDNA assays (ranging from 35–74% and 79–100%, respectively) [2, 33–35]. The specificity of anti-histone autoantibodies for SLE is still under debate, and the observed prevalence (<50%) in SLE patients is always lower compared to anti-dsDNA and anti-chromatin reactivity [36,37], as we have previously described [29]. As outlined above, the large variation in sensitivity and specificity found in different studies can likely be explained by differences in the source and methodology for the isolation of chromatin or histones [2, 23]. Remarkably, we found no correlation for H4p^ac^/H4p and H3p^me^ with the Crithidia and Farr assays, and for H3p with Crithidia assay. Note that previously, we also did not observe a correlation of anti-histone antibodies with Farr or Crithidia [29]. Altogether, we suggest that using apoptosis modified histone peptides offer a reliable alternative with compatible sensitivity and specificity, as these peptides represent the original autoantigen, i.e. apoptotic chromatin, more accurately.

Anti-dsDNA and anti-chromatin reactivity have regularly been associated with disease activity and the occurrence of flares in SLE patients [2, 38–41]. Here, we demonstrate that reactivity with H4p^ac^ and H2Bp^ac^ correlated highly with an increased SLEDAI score and decreased levels of C3, and followed disease activity and anti-dsDNA reactivity during flares in some selected cases we could evaluate. Additionally, an increased SLEDAI was observed in patients reacting with multiple modified peptides. Patients with a high reactivity with these modified peptides also appeared to have an increased number of flares or relapses. Therefore, our results suggest that anti-H4p^ac^ and anti-H2Bp^ac^ reactivity reflects disease activity.

SLE can manifest in multiple organs, including the skin, kidneys, and brain. Therefore, diagnostic markers that relate to the involvement of specific organs could be helpful. We here observed distinct differences in anti-peptide reactivity in SLE patients with nephritis as compared to patients without nephritis. In particular, the ratio of reactivity between modified and unmodified peptide was higher for H2Bp^ac^ in patients with renal involvement. Differences in anti-histone reactivity have occasionally been associated with particular disease manifestations in SLE patients, including neuropsychiatric lupus [37], discoid lupus [42], and incomplete lupus syndromes [43]. Furthermore, anti-chromatin, anti-dsDNA, and anti-histone reactivity are unequivocally correlated to renal involvement [43], while simultaneous positivity for all three markers has been suggested as a possible marker for severe lupus nephritis [44]. However, although anti-chromatin and anti-histone reactivity are related to renal involvement, measuring these reactivities using conventional chromatin or histones as substrates, is not considered to be useful to differentiate SLE with nephritis from SLE without nephritis [45]. Recently, an important role for anti-H2B autoantibodies, and plasma cells producing them, in renal inflammation was reported in the murine NZBWF1 lupus model, although no modified H2B peptides were tested [46]. We found significantly higher anti-H2Bp^ac^ reactivity in SLE patients with renal involvement.

In conclusion, autoantibodies against modified histone peptides H2Bp^ac^ and H4p^ac^ are specific and highly sensitive for SLE patients with lupus nephritis, and correlate with disease activity. Our results emphasize the central role of cell death-associated histone modifications in development of an anti-chromatin autoimmune response in SLE patients that we and others have proposed [4,8–10, 24–25, 47–49]. Obviously, for future application of cell death-associated histone peptides in diagnostic tests, our results would need confirmation by other labs using additional patient and control cohorts.

## Supporting Information

S1 Table Disease characteristics and renal parameters in SLE patients with nephritis divided into quartiles based on their reactivity with H2Bp^ac^ (similar results were observed for H4p^ac^). (PDF)

S2 Table Characteristics of SLE patients experiencing a disease flare shown in Fig 2b. (PDF)
