# Supplementary material for: Autoantibodies against Modified Histone Peptides in SLE Patients Are Associated with Disease Activity and Lupus Nephritis
Source: PLoS One. 2016 Oct 25;11(10):e0165373. doi: 10.1371/journal.pone.0165373 (PMC5079581; doi:10.1371/journal.pone.0165373)
Supplement: S1 Table — (PDF) [file pone.0165373.s001.pdf]

Supplementary data

Table S1. Disease characteristics and renal parameters in SLE patients with nephritis divided into quartiles based on their reactivity with H2Bp^{ac} (similar results were observed for H4p^{ac}).

Quartile	1	2	3	4	whole group
Range (AU)	0-21	21-38	38-83	>83	0-216
n	15	16	15	15	61
age (years)	36.5	32.6	36.5	32.2	34.5
female/male (n)	13/2	14/2	14/1	14/1	55/6
LN First manifestation (n)	7	6	4	9	26
C3 (g/l)	0.65	0.63	0.49	0.43	0.55
C4 (g/l)	0.18	0.13	0.11	**0.12***	0.14
SLEDAI	13.8	13.7	18.0	**21.8***	16.8
Serum creatinine (µM)	139.2	112.8	132.5	118.2	125.7
Glomerular filtration rate	69.2	74.6	68.2	67.0	69.8
Proteinuria (g/24h)	4.31	4.28	3.82	3.80	4.05
Serum creatinine last follow-up (µM)	129.3	88.4	100.6	106.7	106.3
Proteinuria last follow-up (g/24h)	0.52	0.42	0.60	0.24	0.45
Sustained doubling creatinine (n)	1	1	1	3	6
Relapse (n)	2	3	3	4	12

* $p < 0.05$
