# Supplementary material for: Autoantibodies against Modified Histone Peptides in SLE Patients Are Associated with Disease Activity and Lupus Nephritis
Source: PLoS One. 2016 Oct 25;11(10):e0165373. doi: 10.1371/journal.pone.0165373 (PMC5079581; doi:10.1371/journal.pone.0165373)
Supplement: S2 Table — (PDF) [file pone.0165373.s002.pdf]

Table S2. Characteristics of SLE patients experiencing a disease flare shown in Figure 2b.

Patient #	Manifestation at flare	Recent onset disease	Treatment during onset flare
**1**	serositis	no	150 mg azatioprine + 40 mg prednisolone
**2**	vasculitis, arthritis, rash	no	100 mg azatioprine + 8.75 mg prednisolone
**3**	nephritis, leucopenia, rash, myositis	no	1000 mg mycophenolate mofetil + 30 mg prednisolone
**4**	nephritis, vasculitis, rash	no	200 mg HQ + 15 mg prednisolon
